# Clonal hematopoiesis in sickle cell disease

**DOI:** 10.1172/JCI156060

**Published:** 2022-02-15

**Authors:** L. Alexander Liggett, Liam D. Cato, Joshua S. Weinstock, Yingze Zhang, S. Mehdi Nouraie, Mark T. Gladwin, Melanie E. Garrett, Allison Ashley-Koch, Marilyn J. Telen, Brian Custer, Shannon Kelly, Carla L. Dinardo, Ester C. Sabino, Paula Loureiro, Anna B. Carneiro-Proietti, Cláudia Maximo, NHLBI Trans-Omics for Precision Medicine (TOPMed) Consortium, Alexander P. Reiner, Gonçalo R. Abecasis, David A. Williams, Pradeep Natarajan, Alexander G. Bick, Vijay G. Sankaran

**Affiliations:** 1 Division of Hematology/Oncology, Boston Children’s Hospital and Department of Pediatric Oncology, Dana-Farber Cancer Institute, Harvard Medical School, Boston, Massachusetts, USA.; 2 Broad Institute of MIT and Harvard, Cambridge, Massachusetts, USA.; 3 Center for Statistical Genetics, Department of Biostatistics, University of Michigan School of Public Health, Ann Arbor, Michigan, USA.; 4 Department of Medicine, School of Medicine, University of Pittsburgh, Pittsburgh, Pennsylvania, USA.; 5 Department of Medicine, Duke University Medical Center, Durham, North Carolina, USA.; 6 Vitalant Research Institute, San Francisco, California, USA.; 7 Department of Laboratory Medicine, UCSF, San Francisco, California, USA.; 8 Division of Pediatric Hematology, UCSF Benioff Children’s Hospital, Oakland, California, USA.; 9 Fundação Pró-Sangue Hemocentro de São Paulo, São Paulo, Brazil.; 10 Institute of Tropical Medicine, Faculdade de Medicina da Universidade de São Paulo, São Paulo, Brazil.; 11 Fundação Hemope, Recife, Pernambuco, Brazil.; 12 Fundação Hemominas, Belo Horizonte, Brazil.; 13 Fundação Hemorio, Rio de Janeiro, Brazil.; 14 The TOPMed Consortium is detailed in Supplemental Acknowledgments.; 15 Department of Epidemiology, University of Washington, Seattle, Washington, USA.; 16 Division of Public Health Sciences, Fred Hutchinson Cancer Research Center, Seattle, Washington, USA.; 17 Cardiovascular Research Center and Center for Genomic Medicine, Massachusetts General Hospital, Harvard Medical School, Boston, Massachusetts, USA.; 18 Division of Genetic Medicine, Department of Medicine, Vanderbilt University Medical Center, Nashville, Tennessee, USA.; 19 Harvard Stem Cell Institute, Cambridge, Massachusetts, USA.

**Keywords:** Hematology, Leukemias

## Abstract

**BACKGROUND:**

Curative gene therapies for sickle cell disease (SCD) are currently undergoing clinical evaluation. The occurrence of myeloid malignancies in these trials has prompted safety concerns. Individuals with SCD are predisposed to myeloid malignancies, but the underlying causes remain undefined. Clonal hematopoiesis (CH) is a premalignant condition that also confers significant predisposition to myeloid cancers. While it has been speculated that CH may play a role in SCD-associated cancer predisposition, limited data addressing this issue have been reported.

**METHODS:**

Here, we leveraged 74,190 whole-genome sequences to robustly study CH in SCD. Somatic mutation calling methods were used to assess CH in all samples and comparisons between individuals with and without SCD were performed.

**RESULTS:**

While we had sufficient power to detect a greater than 2-fold increased rate of CH, we found no detectable variation in rate or clone properties between individuals affected by SCD and controls. The rate of CH in individuals with SCD was unaltered by hydroxyurea use.

**CONCLUSIONS:**

We did not observe an increased risk for acquiring detectable CH in SCD, at least as measured by whole-genome sequencing. These results should help guide ongoing efforts and further studies that seek to better define the risk factors underlying myeloid malignancy predisposition in SCD and help ensure that curative therapies can be more safely applied.

**FUNDING:**

New York Stem Cell Foundation and the NIH.

## Introduction

In addition to the reduced life expectancy from disease complications (1), individuals with sickle cell disease (SCD) are estimated to have an approximately 3- to 10-fold increased lifetime risk for acquiring acute myeloid leukemia (AML) and other myeloid malignancies (2–4). These observations have recently generated profound interest due to a number of reports of myeloid malignancies arising during gene therapy that have halted several ongoing clinical trials (5, 6). In one reported patient, the malignancy was not attributable to insertional mutagenesis, but the malignant clone was noted to harbor *RUNX1*, *KRAS*, and *PTPN11* mutations (6). While insertional mutagenesis was unlikely the driver of the observed malignancy, it is still possible that other elements of therapy may have contributed to cancer predisposition, such as the conditioning regimen. Analysis of 2 SCD patients with myeloid malignancies in the setting of allogeneic transplantation revealed preexisting hematopoietic clones with *TP53* mutations that increased in size following conditioning, resulting in AML and myelodysplastic syndromes (7). It is still unknown what factors underlie the clonal expansion of these mutant cells, but chronic inflammation and functional decline of tissues may contribute.

The baseline rate of clonal hematopoiesis (CH) in SCD patients and thus the overall risk for myeloid malignancies in these individuals attributable to CH remains unclear. We reasoned that the more frequent and earlier onset of myeloid malignancies in people with SCD would be accompanied by the precocious development of CH. Myeloid malignancy predisposition is generally present in the setting of CH with a variant allele frequency (VAF) above 10% (8–12). Here, we have robustly assessed the prevalence of CH in individuals with SCD who were subject to sequencing and variant calling in tandem with other unaffected individuals as part of the National Heart, Lung and Blood Institute (NHLBI) Trans-Omics for Precision Medicine (TOPMed) consortium to interrogate whether SCD may predispose to CH (13).

## Results

### Prevalence of CH within the SCD population.

To establish the prevalence of CH across human age, whole-genome sequencing (WGS) data from 71,100 individuals unaffected by SCD and 3,090 individuals affected by SCD with ages of 70 years old and below were compiled from 30 distinct cohorts sequenced as part of the TOPMed consortium (Supplemental Figure 1; supplemental material available online with this article; https://doi.org/10.1172/JCI156060DS1). Previously described methods were employed to identify somatic variants and the presence of clonal expansions indicative of CH (14). In short, somatic mutations in the blood were identified by GATK Mutect2 using a panel of normal samples to correct for genomic loci that were hotspots for sequencing artifacts, followed by filtering of identified loci to those that are reliably classified as preleukemic driver mutations (15). Samples were sequenced to an average depth of 38×, which as we have shown previously, detects almost all clones that exist above 10% VAF, and about half of all clones that exist between VAFs of 5% and 10% (14). Although hematopoietic expansions exist in most individuals, and deep sequencing can retroactively identify early origins of malignancies (1, 7), our analysis is sufficient to reliably detect the more mature clones that are of prognostic value based on analyses done to date (11, 12).

**Figure 1:**
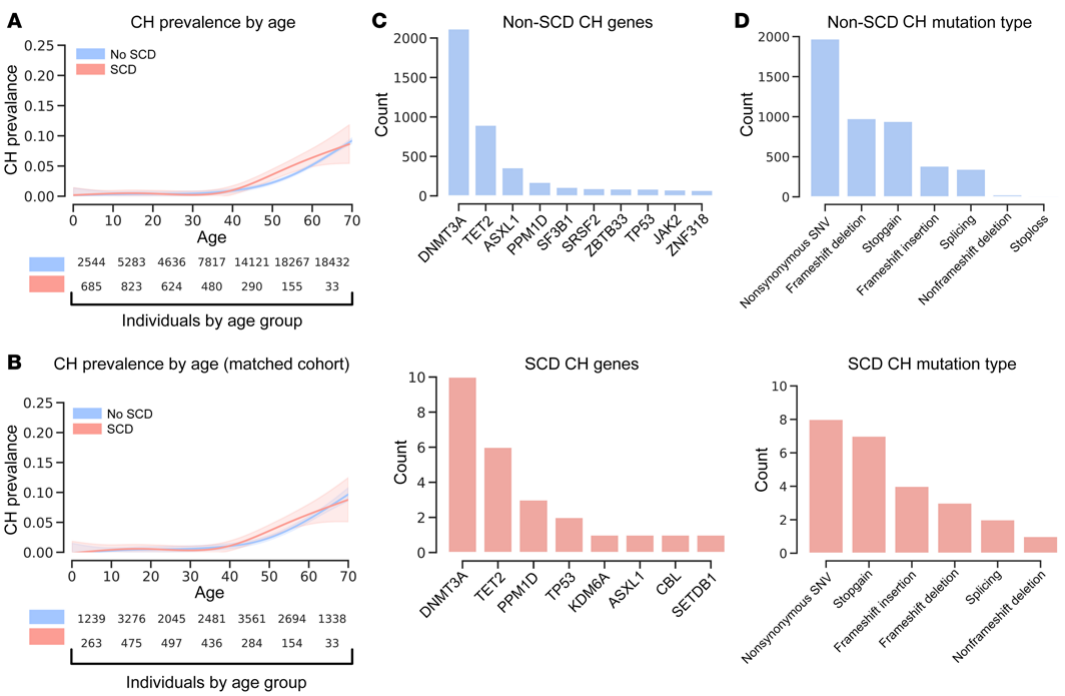
Prevalence of CH is similar in unaffected and SCD populations. (**A**) A generalized additive model was used here to fit rates of CH within WGS data from a total of 71,100 individuals unaffected by SCD and 3,090 individuals affected by SCD, which indicates no significantly increased prevalence of CH within individuals affected by SCD (OR = 1.30, *P =* 0.20). (**B**) Generalized additive model as in **A** was used to fit rates of CH using a genetically matched cohort without SCD. The matched cohort was created by selecting the 10 most similar individuals by the first 10 PCs for each individual in the SCD cohort. Resampling of individuals without SCD was permitted. SCD samples without sufficient matches were excluded. (**C**) Genes ranked by variant load across all individuals separated by SCD status into unaffected (blue) and affected (red). (**D**) Type of genetic change ranked by prevalence in all individuals separated by SCD status into unaffected (blue) and affected (red).

We identified CH in 27 SCD patients and 3,063 unaffected individuals. The prevalence of CH in the SCD cohorts and unaffected individuals strongly correlated with age. There was, however, no significant difference in the prevalence of CH within the SCD cohort when compared to unaffected controls in unadjusted analyses (OR = 1.30, *P =* 0.20; Figure 1A). Using a generalized linear model to account for covariates that are likely to impact the predicted CH prevalence, including age, age^2^, sex, study, and 10 principal components (PCs) of genetic ancestry, we found that the prevalence of CH in individuals with SCD was not elevated over unaffected controls (SCD OR = 1.55, *P =* 0.20; Supplemental Figure 2). As a further sensitivity analysis, we performed a 1:20 case/control matching on age, sex, and the first 10 PCs, as well as excluding several smaller disease-specific cohorts (details in Methods), to identify a group of controls that were as similar as possible to our SCD cases. Even with this constructed set of unaffected controls, the CH prevalence in the SCD cohort was not significantly elevated, and even trended lower (OR = 0.80, *P =* 0.29; Figure 1B).

Although our set of controls included individuals with sickle cell trait (2.6% of controls), we do not observe any altered rate of CH in these individuals compared to the other controls (OR = 1.09, *P =* 0.54; Supplemental Figure 3). Similar subsetting of the SCD cohort to just those individuals with a hemoglobin SS (Hb SS) homozygous genotype, which is the most common SCD genotype, showed no significant difference in prevalence of CH between Hb SS SCD and unaffected individuals (OR = 1.34, *P =* 0.22; Supplemental Figure 4). Furthermore, separating the different Hb genotypes revealed that Hb Sβ^0^ thalassemia was closest to a significantly different odds of CH, although it did not reach significance and only 84 individuals had this genotype (OR = 3.81; *P =* 0.067). Moreover, Hb Sβ^+^ thalassemia (OR = 1.21; *P =* 0.85) and Hb SC disease (OR = 1.02; *P =* 0.97) were both far from significantly different with respect to non-SCD participants. In the absence of explicit annotation of disease severity, the lack of any elevated CH prevalence by genotype, especially for Hb SS which is typically more severe, suggests that there is little impact of disease severity on CH prevalence. Due unfortunately in part to the decreased survival in individuals with SCD, the cohort size diminished with age, but nonetheless with the cohorts available to us, we have 95% power to detect a 2-fold difference and 80% power to detect a 1.75-fold difference in the prevalence of CH in SCD. As the prevalence of CH increases with age, accumulation of additional samples affected by SCD may reveal subtle differences to which we are blind due to the current limited number of individuals.

Tissue microenvironmental and intrinsic conditions have a profound impact on the selective advantages and disadvantages conferred on cells by somatic mutations (16, 17). We therefore expected that while the overall prevalence of CH in SCD may not be elevated, the oncogenic drivers of CH in SCD may notably differ. While categorizing somatic drivers of CH by affected gene and ranking by most prevalent results in slightly different spectra, no genes are significantly more likely to be mutated and represented in expanded clones in the context of SCD (Figure 1C and Supplemental Figure 5). Similarly, classifying somatic mutations by type of change exposes no significant differences in the types of somatic mutations that occur in the context of SCD (Figure 1D and Supplemental Figure 5). By treating the number of CH variants per individual as an ordinal variable and correcting for age in a proportional odds logistic regression, having SCD did not correlate with an increased number of detected mutation-harboring clones (Supplemental Figure 6). The overwhelming majority (>70%) of individuals with CH in our data set had only a single detectable somatic CH mutation, and this did not differ between individuals with SCD and unaffected individuals.

**Figure 2:**
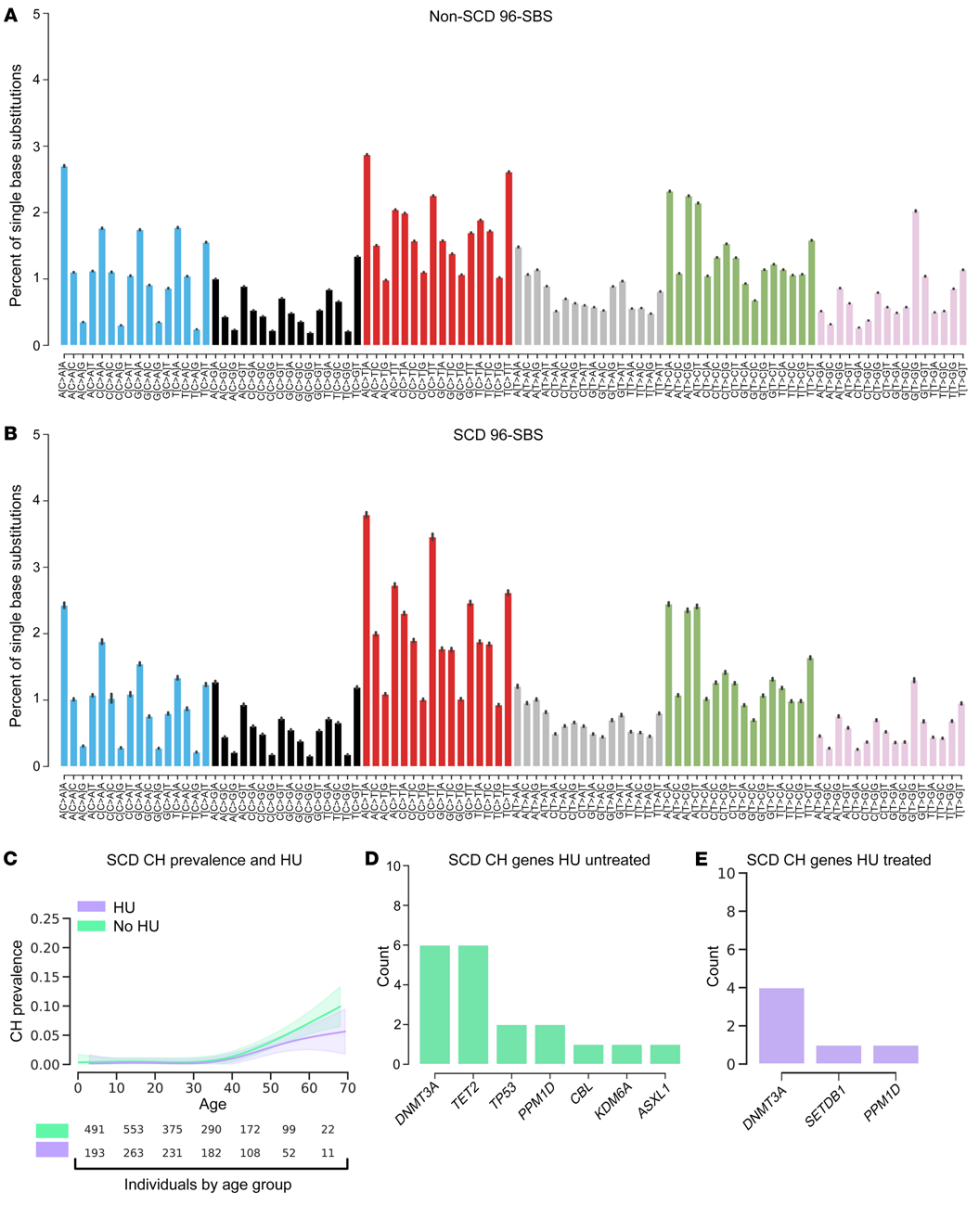
Mutation signatures do not vary in SCD and hydroxyurea (HU) treatment does not impact the rate of CH development. Percentages of total single-base substitutions made up by each possible 96 substitution and trinucleotide context pairs per individual (error is SD across individuals). Signatures are separated into (**A**) individuals without SCD and (**B**) individuals with SCD (data represent mean ± SD). (**C**) A generalized additive model is used here to fit rates of CH within WGS from individuals with SCD separated into never-HU-treated or HU-treated groups. There is no significant difference in the rate of CH in either the HU-treated or untreated groups adjusted for age, age^2^, sex, study, and the first 10 principal components (OR = 0.58, *P =* 0.23). (**D**) Genes ranked by variant load across all individuals with SCD separated by HU treatment status into untreated (green) and (**E**) treated (purple).

While overall rates of CH may not significantly differ among individuals with or without SCD, mutagenic processes may be altered by specific pathogenic features of SCD. We expect that such mutagenic processes could be reflected as alterations to the relative rates of single-base substitutions (SBSs). Importantly, the trinucleotide SBS mutation spectrum for the unaffected individuals showed no significant difference from those individuals with SCD (cosine similarity of 0.98; Figure 2, A and B). This similarity suggests that at least as seen through SBS, there are no prominent mutagenic processes that are uniquely observed or that predominate in the context of SCD.

### Impact of hydroxyurea treatment on the development of CH in SCD.

Hydroxyurea (HU) has been shown to have a significantly positive clinical impact in individuals with SCD and is thought to primarily influence disease severity by increasing levels of fetal Hb (HbF) (18). Given its cytotoxic effects and evidence from animal models, it has been posited that HU may be mutagenic and potentially carcinogenic, despite demonstrated safety profiles in vivo (19). We therefore reasoned that while the overall SCD cohort may not exhibit an elevated risk of developing CH, those treated with HU may. Treating individuals with SCD categorically as either ever having received HU treatment during their clinical course or not, we found no significant elevation in CH prevalence in the group treated with HU (OR = 0.58, *P =* 0.23; Figure 2C). We additionally reasoned that while HU treatment may not drive an apparent increased prevalence of CH, it could select for cells with mutations in a different spectrum of genes. As before, we saw no significant difference in the spectrum of mutated genes in the HU-treated group (Figures 2, D and E, and Supplemental Figure 5). There appeared to be a trend toward a reduced rate of CH in the HU-treated individuals with SCD, but there was no significant difference in individuals over 50 years of age (OR = 0.61; *P =* 0.55). While the data were not available to us, more detailed information such as age, dosage, or duration of treatment would be valuable for additional CH analyses and should be a focus of future studies.

## Discussion

Although not well studied, SCD has been associated in multiple studies with predisposition to myeloid malignancies and this has become of acute interest as the cancers observed in ongoing gene therapy trials have halted several current efforts (4). As part of one of these trials being conducted by the company Bluebird bio, 2 participants enrolled have developed myeloid malignancies (5). While this is only a fraction of the individuals enrolled in the study, it may indicate an elevated risk for malignancies in individuals with SCD. This risk may in part be the result of a preexisting elevated myeloid malignancy risk in individuals with SCD. CH is an important factor predisposing to myeloid malignancies and it has been speculated that individuals with SCD may have elevated rates of CH (20). A preliminary study even reported an observed, albeit slight, increase in CH in people with SCD within a pilot cohort in comparison with external controls (21).

To advance our understanding of CH rates in people with SCD, we have taken advantage of the largest genome sequencing study of individuals with SCD to date and do not observe any increased rate of detectable CH compared to other individuals that were sequenced together with the SCD samples. With the size of the SCD cohorts available to us, we are well powered to detect at least a 2-fold increased prevalence of CH within our current cohorts. Additionally, we detected CH using WGS and were unable to detect extremely small clones. The clinical implications of small clones in predisposing to myeloid malignancies is unknown and while small clones have been retroactively identified after expansion in the context of SCD (1, 7), existing literature suggests that only larger clones with VAFs greater than 10% have prognostic impact (8–11). While further studies are needed, especially to better understand the prognostic value of rare mutations, particularly in the setting of conditioning, transplant, or other treatment regimens, our findings are likely to inform clinical decisions and suggest that CH surveillance as part of routine clinical care for SCD patients is no more indicated at the current time than it is in the general population. In the setting of clinical trials testing curative gene therapy or genome editing approaches, CH surveillance may not provide an effective approach to mitigate malignancy risk. The precise underlying factors responsible for this increased risk will require further study, and may include the chronic stress of hemolysis, an inflammatory bone marrow microenvironment, stressful in vitro cell manipulation, and exposure to cytotoxic conditioning regimens. We believe that the rapid reporting of these results will assist in the ongoing search for the causes of myeloid malignancies in SCD and help define ways to reduce this risk, which is particularly relevant to current and future genetic therapies for this disease.

## Methods

### WGS samples.

Thirty studies from the TOPMed consortium were compiled together into a single data set. The studies were the following: Amish, ARIC, BAGS, BioMe, CARDIA, CFS, CHS, COPDGene, CRA, DHS, FHS, GALAII, GeneSTAR, GenSalt, GOLDN, HCHS_SOL, HyperGEN, JHS, MESA, MLOF, OMG_SCD, REDS-III_Brazil, SAFS, Samoan, SAPPHIRE_asthma, SARP, THRV, VU_AF, walk_PHaSST, and WHI. Together, the studies had 71,100 individuals unaffected by SCD and 3,090 individuals affected by SCD, with a mean of 2,982 individuals per cohort.

### Somatic mutation calling.

Somatic mutations were called from WGS samples using GATK-Mutect2 in combination with GATK-Mutect2-PON (14). Analysis was performed using publicly available methods in workflow description language (WDL) on the Broad Institute’s Terra Platform (https://terra.bio/), and BAM files were remapped and harmonized through a unified protocol. Single-nucleotide polymorphisms (SNPs) and short indels were jointly discovered and genotyped across the TOPMed samples using the GotCloud pipeline (https://genome.sph.umich.edu/wiki/GotCloud). An SVM filter was trained to discriminate between true variants and low-quality sites. Sample quality was assessed through pedigree errors, contamination estimates, and concordance between self-reported sex and genotype-inferred sex. Variants were annotated using SnpEff 4.3 (http://pcingola.github.io/SnpEff/). Putative somatic SNPs and short indels were called with GATK Mutect2 (https://software.broadinstitute.org/gatk). In brief, Mutect2 searches for sites where there is evidence for variation and then performs local reassembly. It uses an external reference of recurrent sequencing artefacts termed a “panel of normal samples” to filter out these sites, and calls variants at sites where there is evidence for somatic variation. The panel of normal samples used for our study included 100 randomly selected individuals under the age of 40 years. Absence of a hotspot CH mutation was verified before inclusion in the panel of normal set. An external reference of germline variants was provided to filter out likely germline calls. We deployed this variant calling process on Google Cloud using Cromwell (https://github.com/broadinstitute/cromwell). The caller was run individually for each sample with the same settings. The Cromwell WDL configuration config files for the run conditions found in this manuscript can be found on the Sankaran lab github (https://github.com/sankaranlab/scd-chip). Variants that appeared within a prespecified list of common driver leukemia driver mutations and passed Mutect2 filters were retained and used to classify individuals as having CH of indeterminate potential (CHIP) (14). Passenger mutations were required to pass Mutect2 filtering, and additionally were only permitted to be found within a single individual within the cohort to minimize the risk of including sequencing or library preparation errors.

### Sickle trait identification.

Individuals were genotyped and called using the GotCloud/vt pipeline in Freeze 8 of TOPMed (https://genome.sph.umich.edu/wiki/Vt, https://genome.sph.umich.edu/wiki/GotCloud). Bcftools was used to extract the rs334 locus. Any individual carrying a heterozygous call (T/A) at that locus was determined a sickle trait carrier.

### CH prevalence modeling.

Somatic variants were retained if they passed Mutect2 filtering, and if they were found within a prespecified list of common leukemia and CHIP driver mutations (14). Other presumably passenger somatic variants that passed Mutect2 filtering were only included in prevalence modeling if they were present within a single individual across all cohorts. All individuals under the age of 70 were included in the study. This cutoff excluded 6 samples affected by SCD, but the limit was chosen to include only those age ranges where there was a sufficient density of samples that a CH prevalence comparison could be made. To derive effect estimates, a binomial logistic regression model was then fit to the data which was annotated for the presence or absence of a clone indicative of CH. Models were then adjusted, where indicated, for age, age^2^, sex, study, sickle cell genotype (Hb genotype driving the SCD), HU use (samples classified as either never having used HU or having used HU for some period of time during their lifetime), and the first 10 PCs. At all instances in the text, ORs are adjusted for at least age and age^2^. Graphically, in most cases, generalized additive models were used to represent patterns across age, with shaded regions corresponding to 95% confidence intervals. Model assumptions were checked in all cases. To incorporate HU treatment, individuals were classified as either undergoing HU treatment or not; treatment was not handled as a continuous variable. To build ideally matched control cohorts for individuals with SCD, the first 10 PCs and age were used to select the 20 most closely matching individuals from the entire unaffected cohort for each individual with SCD, using a nearest-neighbor approach on propensity scores, exact matching was performed on sex. For the matching analysis, smaller disease-specific cohorts (DHS, ECLIPSE, VU_AF, CHS, HVH, GOLDN, Mayo_VTE, EOCOPD, IPF, ECLIPSE, ARIC, and FHS) were excluded. Matching quality was evaluated by Love plots, with a threshold of 0.1 for absolute mean differences as well as visual inspection of each covariate against propensity score, separated by SCD diagnosis. Subsequently, weightings from this matching were used in binomial logistic regression analysis, and pair membership was used to ensure estimated effects and variance were cluster robust. This constructed control cohort was then used as a comparison for CH prevalence as matched controls. For all prevalence studies, the full spectrum of ages was included for all analyses.

### CH gene ranking.

To rank the most commonly mutated genes, singleton variants were filtered to those that are considered to be indicative of the presence of CH. Genes were then ranked by the number of somatic variants they contained across all cohorts.

### Power calculations.

Power calculations were performed using all individuals 70 years of age and younger without SCD as the control population, and using all individuals 70 years of age and younger with SCD as the test population. Power cutoffs were binned at 5% thresholds, and cohort SCD cohort numbers were then used to calculate the maximum bin that powered at a particular fold difference in CH. Across the entire cohort, there was a 4.3% rate of CH, and using all 3,090 individuals with SCD provides at least 95% power to detect a 1.5-fold difference in the rate of CH, requiring a minimum of 1,390 individuals to do so. The probability of type I error (α) used was 0.05. Our SCD cohort was, however, biased toward young individuals, and given that young individuals are typically unlikely to exhibit CH, it is more informative to limit the power calculation to individuals within an older age group that is typically susceptible to developing CH. Within just the range of 40 years of age or older, individuals combined across cohorts without SCD had a 6.78% rate of CH. The experimental population with SCD equal to or above the age of 40 contained 478 individuals. The probability of type I error (α) used was 0.05. In order to detect a 2-fold or 13.56% rate of CH within the experimental SCD population with 95% power, a minimum of 243 individuals are required. To detect a 1.75-fold rate of CH with 80% power, a minimum of 406 individuals are required. We therefore expect that we are at least sufficiently sensitive to detect a 2-fold increase in CH with 95% power and a 1.75-fold increase with 80% power within our entire SCD cohort.

### SBS mutation spectra.

To measure relative rates of SBS in each individual, somatic mutations were first binned by individual and annotated by SCD status. Using hg38 as the reference genome, the neighboring upstream and downstream bases were associated with each somatic substitution mutation to define the trinucleotide SBS for each change. SigProfilerMatrixGenerator was then used to concatenate together each of these trinucleotide changes and compile a full matrix containing all individuals used in this study (22). The relative occurrences of these trinucleotide changes were then calculated per individual and plotted across all individuals with standard deviation as the estimation of error.

### Data availability.

Individual WGS data for TOPMed whole genomes, individual-level harmonized phenotypes, harmonized germline variant call sets, and the CH somatic variant call sets are available through restricted access via the NCBI’s database of Genotypes and Phenotypes (dbGaP). Accession numbers for these data sets have been described in our prior study (14), except for REDS-III_Brazil (phs001468), OMG_SCD (phs001608), and walk_PHaSST (phs001514). Deidentified variant calls are available from the authors upon request.

### Statistics.

Generalized linear models used to compare age-related differences in CH between non-SCD and SCD groups used 95% confidence intervals and significance cutoffs of *P* less than 0.05. Generalized additive models (GAMs) were used to graphically represent the data of SCD versus no SCD in Figure 1, A and B, and HU treatment versus no HU in Figure 2C. Logistic regression models produced the reported ORs and *P* values in the manuscript text. To go further, GAMs provide a flexible approach to modeling that accounts for multiple nonlinear effects. All residuals and model parameters were checked and confirmed, and smoothers were checked for overcomplexity. For the logistic regression model outputs reported in the text, *P* values are a result of Wald testing of the model coefficient.

### Study approval.

Written informed consent was obtained from all human participants by each of the studies that contributed to TOPMed with approval of study protocols by ethics committees at participating institutions. All relevant ethics committees approved this study and this work is compliant with all relevant ethical regulations.

## Author contributions

LAL, LDC, JSW, AGB, and VGS conceived and designed the study, and wrote the manuscript with input from all authors. LAL, LDC, and JSW analyzed data. YZ directed the Walk-PHasst repository and SMN maintained the Walk-PHaSST database. YZ, SMN, MTG, MEG, AAK, MJT, BC, SK, CLD, ECS, PL, ABCP, CM, APR, GRA, DAW, and PN contributed clinical data or analytic advice. AGB and VGS supervised the study.

## Supplementary Material

Supplemental data

ICMJE disclosure forms

## Version 1 01/06/2022

In-Press Preview

## Version 2 02/15/2022

Electronic publication
