# Supplementary material for: Clonal hematopoiesis in sickle cell disease
Source: J Clin Invest. 2022 Feb 15;132(4):e156060. doi: 10.1172/JCI156060 (PMC8843701; doi:10.1172/JCI156060)
Supplement: Supplemental data [file jci-132-156060-s095.pdf]

Supplemental Figure 1

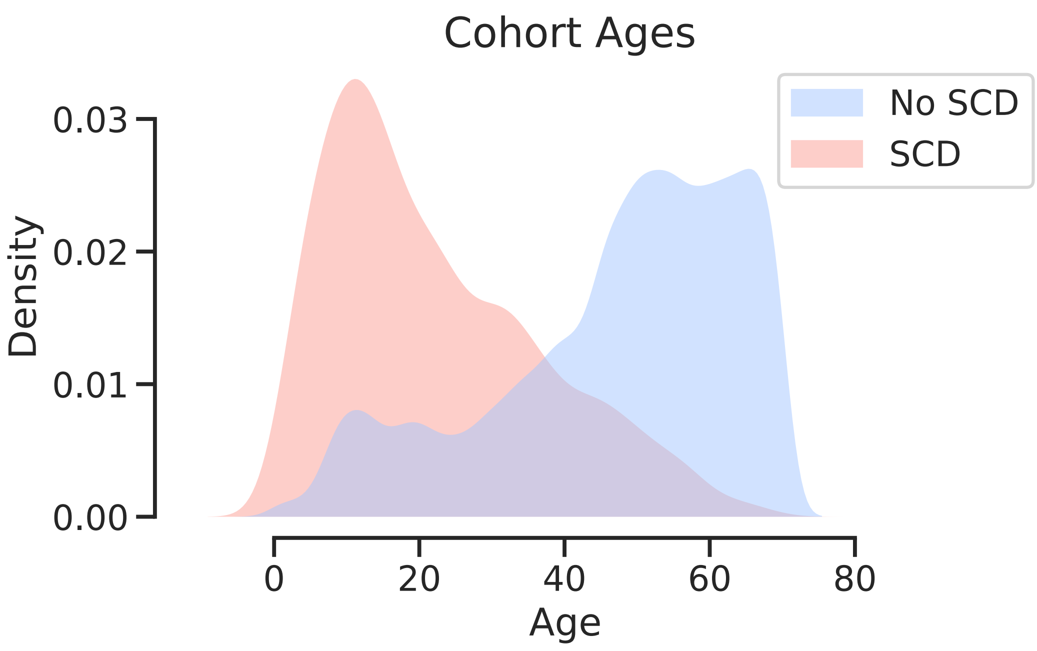

Supplemental Figure 2

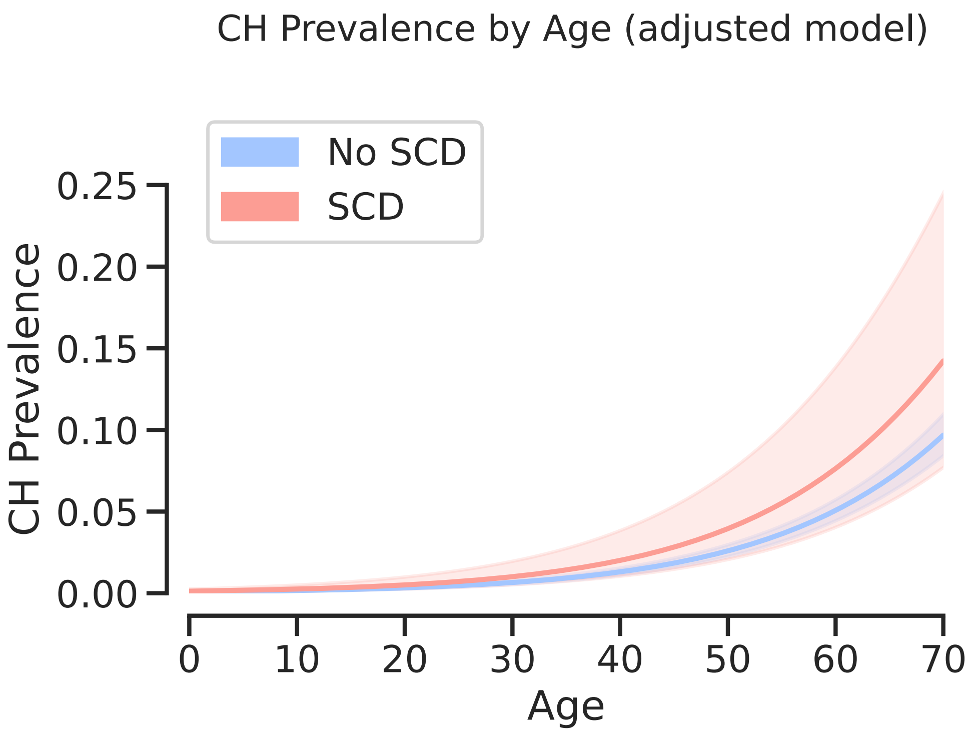

Supplemental Figure 3

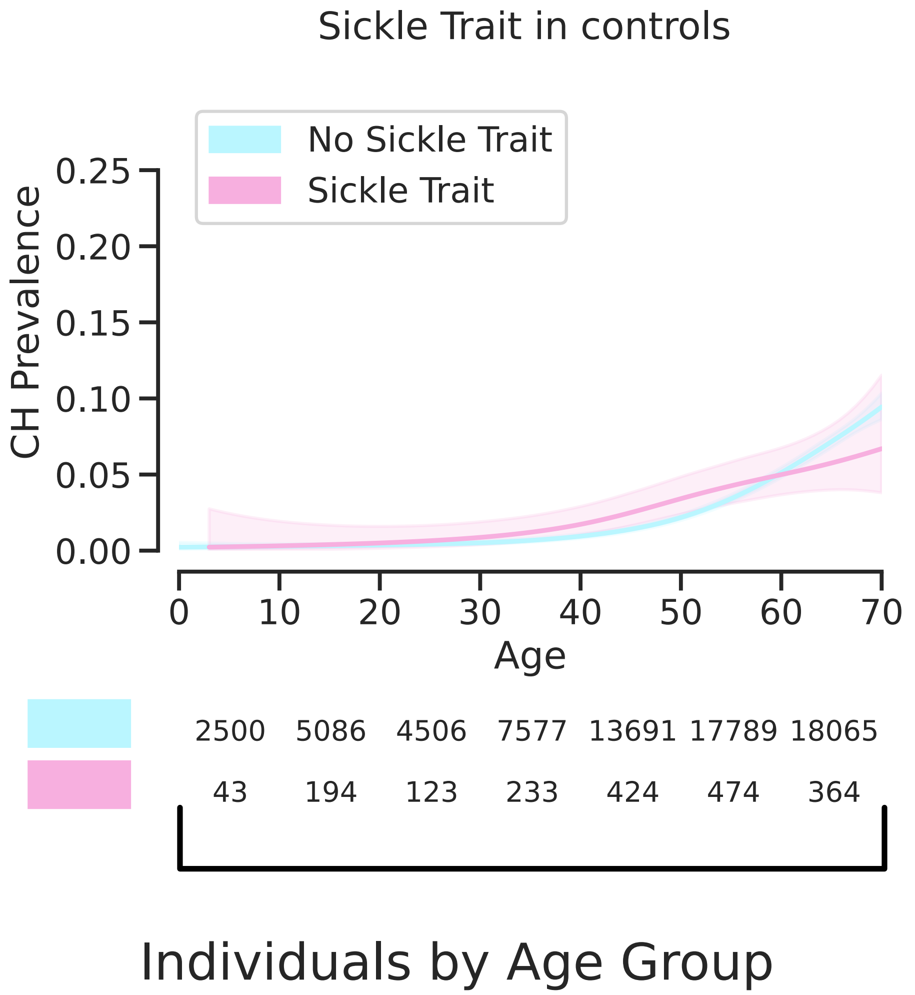

Supplemental Figure 4

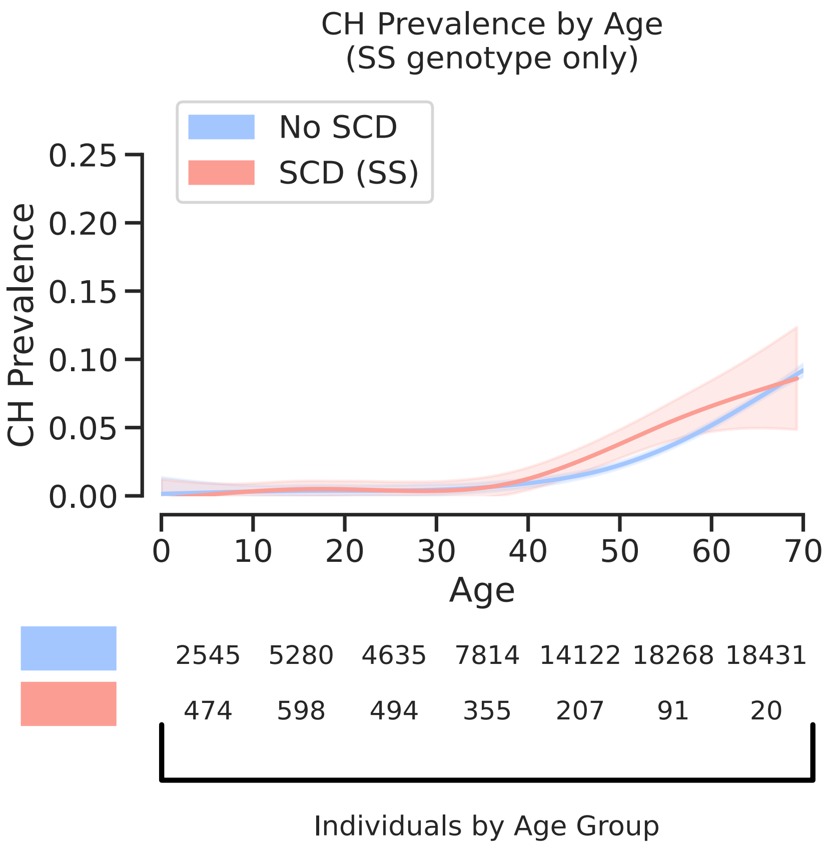

Supplemental Figure 5

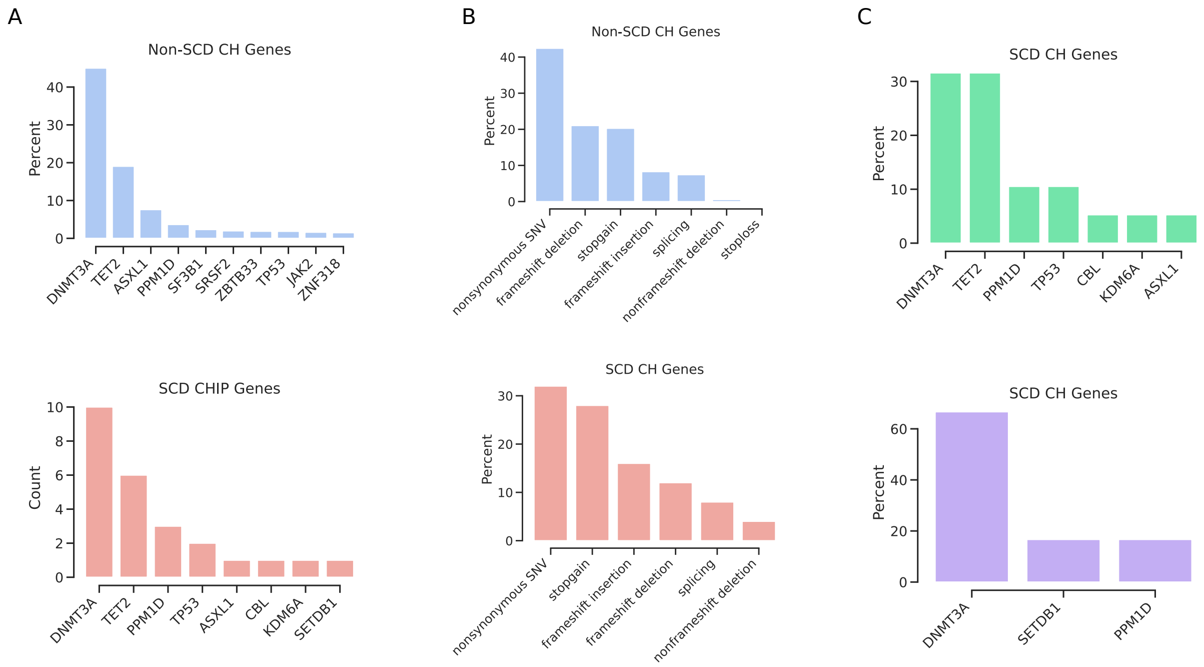

Supplemental Figure 6

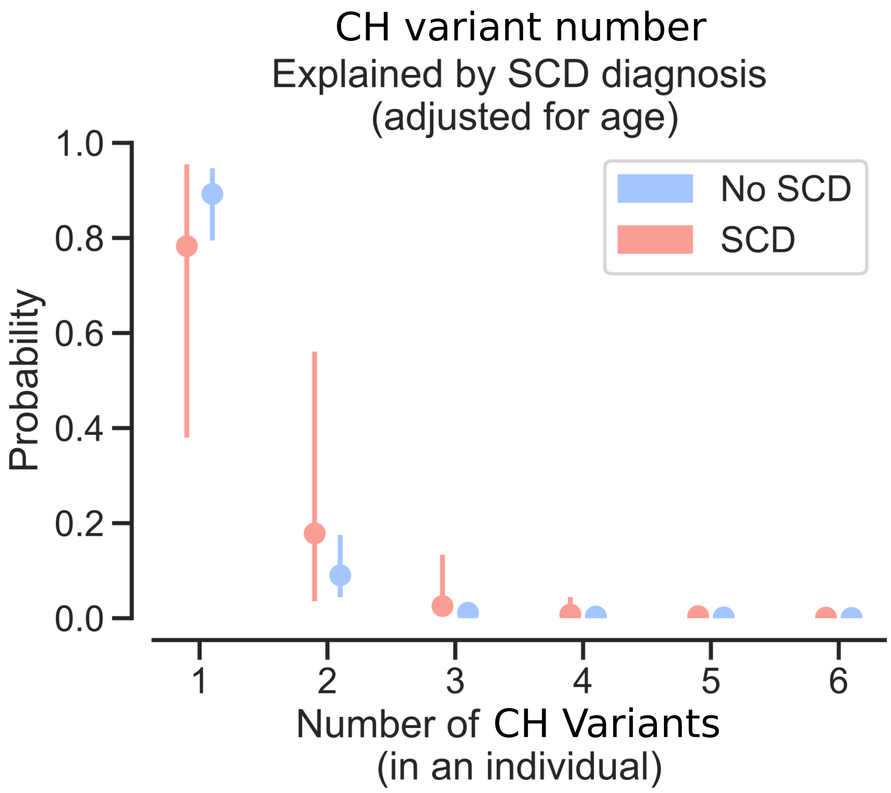

Supplemental Figure 1. Cohort age distributions. Age distributions of all individuals combined from each of the TOPMed cohorts used in this study, separated by SCD status into those without SCD (blue) and those with SCD (red).

Supplemental Figure 2. Matched background displays similar prevalence of CH as SCD cohort. 20 matched controls for each individual with SCD by matching the first 10 PCs, age, and

sex, were pooled together to create a tailored unaffected cohort to compare to SCD affected individuals (OR=0.74, p=0.155).

Supplemental Figure 3. CH prevalence when accounting for sickle cell trait. From the group of all control individuals, those individuals who were heterozygous for the sickle cell trait (rs334) SNP are classified as having the sickle cell trait (pink) and those without it are classified as having no sickle cell trait (blue). Even when accounting for the rs334 SNP, there is no significant difference between the two groups.

Supplemental Figure 4. CH prevalence in SS SCD subgroup. Homozygous SS SCD genotypes alone do not show elevated prevalence of CH compared with unaffected individuals (OR=1.34, p=0.22).

Supplemental Figure 5. (**A**) Genes ranked by variant load across all individuals separated by SCD status into unaffected (blue) and affected (red). (**B**) Type of genetic change ranked by prevalence in all individuals separated by SCD status into unaffected (blue) and affected (red). (**D**) Genes ranked by variant load across all individuals with SCD separated by HU treatment status into untreated (green) and treated (purple).

Supplemental Figure 6. CH clone load per individual is unchanged by SCD. Number of variants per individual classified as indicative of CH appear at similar rates in unaffected individuals and individuals with SCD.

TOPMed Consortium

Details provided here for TOPMed are available at https://topmed.nhlbi.nih.gov/topmed-banner-authorship. The TOPMed banner, NHLBI Trans-Omics for Precision Medicine (TOPMed) Consortium, is used to acknowledge individuals who contributed to the overall conduct of TOPMed but do not otherwise meet criteria for by-line authorship for a given manuscript:

Name	Institution(s)
Abe, Namiko	New York Genome Center
Abecasis, Gonçalo	University of Michigan
Aguet, Francois	Broad Institute
Albert, Christine	Cedars Sinai
Almasy, Laura	Children's Hospital of Philadelphia, University of Pennsylvania
Alonso, Alvaro	Emory University
Ament, Seth	University of Maryland
Anderson, Peter	University of Washington
Anugu, Pramod	University of Mississippi
Applebaum-Bowden, Deborah	National Institutes of Health
Ardlie, Kristin	Broad Institute
Arking, Dan	Johns Hopkins University
Arnett, Donna K	University of Kentucky
Ashley-Koch, Allison	Duke University
Aslibekyan, Stella	University of Alabama
Assimes, Tim	Stanford University
Auer, Paul	Medical College of Wisconsin
Avramopoulos, Dimitrios	Johns Hopkins University
Ayas, Najib	Providence Health Care
Balasubramanian, Adithya	Baylor College of Medicine Human Genome Sequencing Center
Barnard, John	Cleveland Clinic
Barnes, Kathleen	Tempus, University of Colorado Anschutz Medical Campus
Barr, R. Graham	Columbia University
Barron-Casella, Emily	Johns Hopkins University
Barwick, Lucas	The Emmes Corporation
Beaty, Terri	Johns Hopkins University
Beck, Gerald	Cleveland Clinic
Becker, Diane	Johns Hopkins University

Becker, Lewis	Johns Hopkins University
Beer, Rebecca	National Heart, Lung, and Blood Institute, National Institutes of Health
Beitelshees, Amber	University of Maryland
Benjamin, Emelia	Boston University, Massachusetts General Hospital
Benos, Takis	University of Pittsburgh
Bezerra, Marcos	Fundação de Hematologia e Hemoterapia de Pernambuco - Hemope
Bielak, Larry	University of Michigan
Bis, Joshua	University of Washington
Blackwell, Thomas	University of Michigan
Blangero, John	University of Texas Rio Grande Valley School of Medicine
Boerwinkle, Eric	University of Texas Health at Houston
Bowden, Donald W.	Wake Forest Baptist Health
Bowler, Russell	National Jewish Health
Brody, Jennifer	University of Washington
Broeckel, Ulrich	Medical College of Wisconsin
Broome, Jai	University of Washington
Brown, Deborah	University of Texas Health at Houston
Bunting, Karen	New York Genome Center
Burchard, Esteban	University of California, San Francisco
Bustamante, Carlos	Stanford University
Buth, Erin	University of Washington
Cade, Brian	Brigham & Women's Hospital
Cardwell, Jonathan	University of Colorado at Denver
Carey, Vincent	Brigham & Women's Hospital
Carrier, Julie	University of Montreal
Carson, April	University of Mississippi
Carty, Cara	Washington State University
Casaburi, Richard	University of California, Los Angeles
Casas Romero, Juan P	Brigham & Women's Hospital
Casella, James	Johns Hopkins University
Castaldi, Peter	Brigham & Women's Hospital
Chaffin, Mark	Broad Institute
Chang, Christy	University of Maryland
Chang, Yi-Cheng	National Taiwan University
Chasman, Daniel	Brigham & Women's Hospital
Chavan, Sameer	University of Colorado at Denver
Chen, Bo-Juen	New York Genome Center
Chen, Wei-Min	University of Virginia

Chen, Yii-Der Ida	Lundquist Institute
Cho, Michael	Brigham & Women's Hospital
Choi, Seung Hoan	Broad Institute
Chuang, Lee-Ming	National Taiwan University
Chung, Mina	Cleveland Clinic
Chung, Ren-Hua	National Health Research Institute Taiwan
Clish, Clary	Broad Institute
Comhair, Suzy	Cleveland Clinic
Conomos, Matthew	University of Washington
Cornell, Elaine	University of Vermont
Correa, Adolfo	University of Mississippi
Crandall, Carolyn	University of California, Los Angeles
Crapo, James	National Jewish Health
Cupples, L. Adrienne	Boston University
Curran, Joanne	University of Texas Rio Grande Valley School of Medicine
Curtis, Jeffrey	University of Michigan
Custer, Brian	Vitalant Research Institute
Damcott, Coleen	University of Maryland
Darbar, Dawood	University of Illinois at Chicago
David, Sean	University of Chicago
Davis, Colleen	University of Washington
Daya, Michelle	University of Colorado at Denver
de Andrade, Mariza	Mayo Clinic
de las Fuentes, Lisa	Washington University in St Louis
de Vries, Paul	University of Texas Health at Houston
DeBaun, Michael	Vanderbilt University
Deka, Ranjan	University of Cincinnati
DeMeo, Dawn	Brigham & Women's Hospital
Devine, Scott	University of Maryland
Dinh, Huyen	Baylor College of Medicine Human Genome Sequencing Center
Doddapaneni, Harsha	Baylor College of Medicine Human Genome Sequencing Center
Duan, Qing	University of North Carolina
Dugan-Perez, Shannon	Baylor College of Medicine Human Genome Sequencing Center
Duggirala, Ravi	University of Texas Rio Grande Valley School of Medicine
Durda, Jon Peter	University of Vermont
Dutcher, Susan K.	Washington University in St Louis
Eaton, Charles	Brown University

Ekunwe, Lynette	University of Mississippi
El Boueiz, Adel	Harvard University
Ellinor, Patrick	Massachusetts General Hospital
Emery, Leslie	University of Washington
Erzurum, Serpil	Cleveland Clinic
Farber, Charles	University of Virginia
Farek, Jesse	Baylor College of Medicine Human Genome Sequencing Center
Fingerlin, Tasha	National Jewish Health
Flickinger, Matthew	University of Michigan
Fornage, Myriam	University of Texas Health at Houston
Franceschini, Nora	University of North Carolina
Frazar, Chris	University of Washington
Fu, Mao	University of Maryland
Fullerton, Stephanie M.	University of Washington
Fulton, Lucinda	Washington University in St Louis
Gabriel, Stacey	Broad Institute
Gan, Weiniu	National Heart, Lung, and Blood Institute, National Institutes of Health
Gao, Shanshan	University of Colorado at Denver
Gao, Yan	University of Mississippi
Gass, Margery	Fred Hutchinson Cancer Research Center
Geiger, Heather	New York Genome Center
Gelb, Bruce	Icahn School of Medicine at Mount Sinai
Geraci, Mark	University of Pittsburgh
Germer, Soren	New York Genome Center
Gerszten, Robert	Beth Israel Deaconess Medical Center
Ghosh, Auyon	Brigham & Women's Hospital
Gibbs, Richard	Baylor College of Medicine Human Genome Sequencing Center
Gignoux, Chris	Stanford University
Gladwin, Mark	University of Pittsburgh
Glahn, David	Boston Children's Hospital, Harvard Medical School
Gogarten, Stephanie	University of Washington
Gong, Da-Wei	University of Maryland
Goring, Harald	University of Texas Rio Grande Valley School of Medicine
Graw, Sharon	University of Colorado Anschutz Medical Campus
Gray, Kathryn J.	Mass General Brigham
Grine, Daniel	University of Colorado at Denver
Gross, Colin	University of Michigan

Gu, C. Charles	Washington University in St Louis
Guan, Yue	University of Maryland
Guo, Xiuqing	Lundquist Institute
Gupta, Namrata	Broad Institute
Haas, David M.	Indiana University
Haessler, Jeff	Fred Hutchinson Cancer Research Center
Hall, Michael	University of Mississippi
Han, Yi	Baylor College of Medicine Human Genome Sequencing Center
Hanly, Patrick	University of Calgary
Harris, Daniel	University of Maryland
Hawley, Nicola L.	Yale University
He, Jiang	Tulane University
Heavner, Ben	University of Washington
Heckbert, Susan	University of Washington
Hernandez, Ryan	University of California, San Francisco
Herrington, David	Wake Forest Baptist Health
Hersh, Craig	Brigham & Women's Hospital
Hidalgo, Bertha	University of Alabama
Hixson, James	University of Texas Health at Houston
Hobbs, Brian	Brigham & Women's Hospital
Hokanson, John	University of Colorado at Denver
Hong, Elliott	University of Maryland
Hoth, Karin	University of Iowa
Hsiung, Chao (Agnes)	National Health Research Institute Taiwan
Hu, Jianhong	Baylor College of Medicine Human Genome Sequencing Center
Hung, Yi-Jen	Tri-Service General Hospital National Defense Medical Center
Huston, Haley	Blood Works Northwest
Hwu, Chii Min	Taichung Veterans General Hospital Taiwan
Irvin, Marguerite Ryan	University of Alabama
Jackson, Rebecca	Oklahoma State University Medical Center
Jain, Deepti	University of Washington
Jaquish, Cashell	National Heart, Lung, and Blood Institute, National Institutes of Health
Johnsen, Jill	Blood Works Northwest
Johnson, Andrew	National Heart, Lung, and Blood Institute, National Institutes of Health
Johnson, Craig	University of Washington

Johnston, Rich	Emory University
Jones, Kimberly	Johns Hopkins University
Kang, Hyun Min	University of Michigan
Kaplan, Robert	Albert Einstein College of Medicine
Kardia, Sharon	University of Michigan
Kelly, Shannon	University of California, San Francisco
Kenny, Eimear	Icahn School of Medicine at Mount Sinai
Kessler, Michael	University of Maryland
Khan, Alyna	University of Washington
Khan, Ziad	Baylor College of Medicine Human Genome Sequencing Center
Kim, Wonji	Harvard University
Kimoff, John	McGill University
Kinney, Greg	University of Colorado at Denver
Konkle, Barbara	Blood Works Northwest
Kooperberg, Charles	Fred Hutchinson Cancer Research Center
Kramer, Holly	Loyola University
Lange, Christoph	Harvard School of Public Health
Lange, Ethan	University of Colorado at Denver
Lange, Leslie	University of Colorado at Denver
Laurie, Cathy	University of Washington
Laurie, Cecelia	University of Washington
LeBoff, Meryl	Brigham & Women's Hospital
Lee, Jiwon	Brigham & Women's Hospital
Lee, Sandra	Baylor College of Medicine Human Genome Sequencing Center
Lee, Wen-Jane	Taichung Veterans General Hospital Taiwan
LeFaive, Jonathon	University of Michigan
Levine, David	University of Washington
Levy, Dan	National Heart, Lung, and Blood Institute, National Institutes of Health
Lewis, Joshua	University of Maryland
Li, Xiaohui	Lundquist Institute
Li, Yun	University of North Carolina
Lin, Henry	Lundquist Institute
Lin, Honghuang	Boston University
Lin, Xihong	Harvard School of Public Health
Liu, Simin	Brown University
Liu, Yongmei	Duke University
Liu, Yu	Stanford University

Loos, Ruth J.F.	Icahn School of Medicine at Mount Sinai
Lubitz, Steven	Massachusetts General Hospital
Lunetta, Kathryn	Boston University
Luo, James	National Heart, Lung, and Blood Institute, National Institutes of Health
Magalang, Ulysses	Ohio State University
Mahaney, Michael	University of Texas Rio Grande Valley School of Medicine
Make, Barry	Johns Hopkins University
Manichaikul, Ani	University of Virginia
Manning, Alisa	Broad Institute, Harvard University, Massachusetts General Hospital
Manson, JoAnn	Brigham & Women's Hospital
Martin, Lisa	George Washington University
Marton, Melissa	New York Genome Center
Mathai, Susan	University of Colorado at Denver
Mathias, Rasika	Johns Hopkins University
May, Susanne	University of Washington
McArdle, Patrick	University of Maryland
McDonald, Merry-Lynn	University of Alabama
McFarland, Sean	Harvard University
McGarvey, Stephen	Brown University
McGoldrick, Daniel	University of Washington
McHugh, Caitlin	University of Washington
McNeil, Becky	RTI International
Mei, Hao	University of Mississippi
Meigs, James	Massachusetts General Hospital
Menon, Vipin	Baylor College of Medicine Human Genome Sequencing Center
Mestroni, Luisa	University of Colorado Anschutz Medical Campus
Metcalf, Ginger	Baylor College of Medicine Human Genome Sequencing Center
Meyers, Deborah A	University of Arizona
Mignot, Emmanuel	Stanford University
Mikulla, Julie	National Heart, Lung, and Blood Institute, National Institutes of Health
Min, Nancy	University of Mississippi
Minear, Mollie	National Institute of Child Health and Human Development, National Institutes of Health
Minster, Ryan L	University of Pittsburgh
Mitchell, Braxton D.	University of Maryland

Moll, Matt	Brigham & Women's Hospital
Momin, Zeineen	Baylor College of Medicine Human Genome Sequencing Center
Montasser, May E.	University of Maryland
Montgomery, Courtney	Oklahoma Medical Research Foundation
Muzny, Donna	Baylor College of Medicine Human Genome Sequencing Center
Mychaleckyj, Josyf C	University of Virginia
Nadkarni, Girish	Icahn School of Medicine at Mount Sinai
Naik, Rakhi	Johns Hopkins University
Naseri, Take	Ministry of Health, Government of Samoa
Natarajan, Pradeep	Broad Institute
Nekhai, Sergei	Howard University
Nelson, Sarah C.	University of Washington
Neltner, Bonnie	University of Colorado at Denver
Nessner, Caitlin	Baylor College of Medicine Human Genome Sequencing Center
Nickerson, Deborah	University of Washington
Nkechinyere, Osuji	Baylor College of Medicine Human Genome Sequencing Center
North, Kari	University of North Carolina
O'Connell, Jeff	University of Maryland
O'Connor, Tim	University of Maryland
Ochs-Balcom, Heather	University at Buffalo
Okwuonu, Geoffrey	Baylor College of Medicine Human Genome Sequencing Center
Pack, Allan	University of Pennsylvania
Paik, David T.	Stanford University
Palmer, Nicholette	Wake Forest Baptist Health
Pankow, James	University of Minnesota
Papanicolaou, George	National Heart, Lung, and Blood Institute, National Institutes of Health
Parker, Cora	RTI International
Peloso, Gina	Boston University
Peralta, Juan Manuel	University of Texas Rio Grande Valley School of Medicine
Perez, Marco	Stanford University
Perry, James	University of Maryland
Peters, Ulrike	Fred Hutchinson Cancer Research Center
Peyser, Patricia	University of Michigan
Phillips, Lawrence S	Emory University

Pleiness, Jacob	University of Michigan
Pollin, Toni	University of Maryland
Post, Wendy	Johns Hopkins University
Powers Becker, Julia	University of Colorado at Denver
Preethi Boorgula, Meher	University of Colorado at Denver
Preuss, Michael	Icahn School of Medicine at Mount Sinai
Psaty, Bruce	University of Washington
Qasba, Pankaj	National Heart, Lung, and Blood Institute, National Institutes of Health
Qiao, Dandi	Brigham & Women's Hospital
Qin, Zhaohui	Emory University
Rafaels, Nicholas	University of Colorado at Denver
Raffield, Laura	University of North Carolina
Rajendran, Mahitha	Baylor College of Medicine Human Genome Sequencing Center
Ramachandran, Vasan S.	Boston University
Rao, D.C.	Washington University in St Louis
Rasmussen-Torvik, Laura	Northwestern University
Ratan, Aakrosh	University of Virginia
Redline, Susan	Brigham & Women's Hospital
Reed, Robert	University of Maryland
Reeves, Catherine	New York Genome Center
Regan, Elizabeth	National Jewish Health
Reiner, Alex	Fred Hutchinson Cancer Research Center, University of Washington
Reupena, Muagututi‘a Sefuiva	Lutia I Puava Ae Mapu I Fagalele
Rice, Ken	University of Washington
Rich, Stephen	University of Virginia
Robillard, Rebecca	University of Ottawa
Robine, Nicolas	New York Genome Center
Roden, Dan	Vanderbilt University
Roselli, Carolina	Broad Institute
Rotter, Jerome	Lundquist Institute
Ruczinski, Ingo	Johns Hopkins University
Runnels, Alexi	New York Genome Center
Russell, Pamela	University of Colorado at Denver
Ruuska, Sarah	Blood Works Northwest

Ryan, Kathleen	University of Maryland
Sabino, Ester Cerdeira	Universidade de Sao Paulo
Saleheen, Danish	Columbia University
Salimi, Shabnam	University of Maryland
Salvi, Sejal	Baylor College of Medicine Human Genome Sequencing Center
Salzberg, Steven	Johns Hopkins University
Sandow, Kevin	Lundquist Institute
Sankaran, Vijay G.	Harvard University
Santibanez, Jireh	Baylor College of Medicine Human Genome Sequencing Center
Schwander, Karen	Washington University in St Louis
Schwartz, David	University of Colorado at Denver
Sciurba, Frank	University of Pittsburgh
Seidman, Christine	Harvard Medical School
Seidman, Jonathan	Harvard Medical School
Sériès, Frédéric	Université Laval
Sheehan, Vivien	Emory University
Sherman, Stephanie L.	Emory University
Shetty, Amol	University of Maryland
Shetty, Aniket	University of Colorado at Denver
Sheu, Wayne Hui-Heng	Taichung Veterans General Hospital Taiwan
Shoemaker, M. Benjamin	Vanderbilt University
Silver, Brian	UMass Memorial Medical Center
Silverman, Edwin	Brigham & Women's Hospital
Skomro, Robert	University of Saskatchewan
Smith, Albert Vernon	University of Michigan
Smith, Jennifer	University of Michigan
Smith, Josh	University of Washington
Smith, Nicholas	University of Washington
Smith, Tanja	New York Genome Center
Smoller, Sylvia	Albert Einstein College of Medicine
Snively, Beverly	Wake Forest Baptist Health
Snyder, Michael	Stanford University
Sofer, Tamar	Brigham & Women's Hospital
Sotoodehnia, Nona	University of Washington
Stilp, Adrienne M.	University of Washington
Storm, Garrett	University of Colorado at Denver
Streeten, Elizabeth	University of Maryland

Su, Jessica Lasky	Brigham & Women's Hospital
Sung, Yun Ju	Washington University in St Louis
Sylvia, Jody	Brigham & Women's Hospital
Szpiro, Adam	University of Washington
Taliun, Daniel	University of Michigan
Tang, Hua	Stanford University
Taub, Margaret	Johns Hopkins University
Taylor, Kent D.	Lundquist Institute
Taylor, Matthew	University of Colorado Anschutz Medical Campus
Taylor, Simeon	University of Maryland
Telen, Marilyn	Duke University
Thornton, Timothy A.	University of Washington
Threlkeld, Machiko	University of Washington
Tinker, Lesley	Fred Hutchinson Cancer Research Center
Tirschwell, David	University of Washington
Tishkoff, Sarah	University of Pennsylvania
Tiwari, Hemant	University of Alabama
Tong, Catherine	University of Washington
Tracy, Russell	University of Vermont
Tsai, Michael	University of Minnesota
Vaidya, Dhananjay	Johns Hopkins University
Van Den Berg, David	University of Southern California
VandeHaar, Peter	University of Michigan
Vrieze, Scott	University of Minnesota
Walker, Tarik	University of Colorado at Denver
Wallace, Robert	University of Iowa
Walts, Avram	University of Colorado at Denver
Wang, Fei Fei	University of Washington
Wang, Heming	Brigham & Women's Hospital, Mass General Brigham
Wang, Jiongming	University of Michigan
Watson, Karol	University of California, Los Angeles
Watt, Jennifer	Baylor College of Medicine Human Genome Sequencing Center
Weeks, Daniel E.	University of Pittsburgh
Weinstock, Joshua	University of Michigan
Weir, Bruce	University of Washington
Weiss, Scott T	Brigham & Women's Hospital
Weng, Lu-Chen	Massachusetts General Hospital
Wessel, Jennifer	Indiana University
Willer, Cristen	University of Michigan

Williams, Kayleen	University of Washington
Williams, L. Keoki	Henry Ford Health System
Wilson, Carla	Brigham & Women's Hospital
Wilson, James	Beth Israel Deaconess Medical Center
Winterkorn, Lara	New York Genome Center
Wong, Quenna	University of Washington
Wu, Joseph	Stanford University
Xu, Huichun	University of Maryland
Yanek, Lisa	Johns Hopkins University
Yang, Ivana	University of Colorado at Denver
Yu, Ketian	University of Michigan
Zekavat, Seyedeh Maryam	Broad Institute
Zhang, Yingze	University of Pittsburgh
Zhao, Snow Xueyan	National Jewish Health
Zhao, Wei	University of Michigan
Zhu, Xiaofeng	Case Western Reserve University
Zody, Michael	New York Genome Center
Zoellner, Sebastian	University of Michigan

Walk-PHaSST

We also thank the Walk-PHaSST clinical site team: Jane Little and Verlene Davis (Albert Einstein College of Medicine); Robyn Barst, Erika Rosenzweig, Margaret Lee, and Daniela Brady (Columbia University); Claudia Morris, Ward Hagar, Lisa Lavrisha, Howard Rosenfeld, and Elliott Vichinsky (UCSF Benioff Children's Hospital Oakland); Regina McCollum (Children's Hospital of Pittsburgh of UPMC); Sally Davies, Gaia Mahalingam, Sharon Meehan, Ofelia Lebanto, and Ines Cabrita (Hammersmith Hospital, London); Victor Gordeuk, Oswaldo Castro, Onyinye Onyekwere, Vandana Sachdev, Alvin Thomas, Gladys Onojobi, Sharmin Diaz, Margaret Fadojutimi-Akinsiku, and Randa Aladdin (Howard University); Reda Girgis, Sophie Lanzkron, and Durrant Barasa (Johns Hopkins University); Mark Gladwin, Greg Kato, James Taylor, Wynona Coles, Catherine Seamon, Mary Hall, Amy Chi, Cynthia Brenneman, Wen Li, and Erin Smith (NHLBI); Kathryn Hassell, David Badesch, Deb McCollister and Julie McAfee (University of Colorado); Dean Schraufnagel, Robert Molokie, George Kondos, Patricia Cole-Saffold, and Lani Krauz (University of Illinois at Chicago); and Simon Gibbs (National Heart & Lung Institute, Imperial College London).
